# Influence of Fiber Orientation on Single-Point Cutting Fracture Behavior of Carbon-Fiber/Epoxy Prepreg Sheets

**DOI:** 10.3390/ma8105336

**Published:** 2015-10-02

**Authors:** Yingying Wei, Qinglong An, Xiaojiang Cai, Ming Chen, Weiwei Ming

**Affiliations:** School of Mechanical Engineering, Shanghai Jiao Tong University, Shanghai 200240, China; weiyingying86@gmail.com (Y.W.); qlan@sjtu.edu.cn (Q.A.); xjiang.cai@gmail.com (X.C.); mchen@sjtu.edu.cn (M.C.)

**Keywords:** fiber orientation, carbon-fiber/epoxy prepreg, single-point flying cutting, fracture morphology, acoustic emission inspection

## Abstract

The purpose of this article is to investigate the influences of carbon fibers on the fracture mechanism of carbon fibers both in macroscopic view and microscopic view by using single-point flying cutting method. Cutting tools with three different materials were used in this research, namely, PCD (polycrystalline diamond) tool, CVD (chemical vapor deposition) diamond thin film coated carbide tool and uncoated carbide tool. The influence of fiber orientation on the cutting force and fracture topography were analyzed and conclusions were drawn that cutting forces are not affected by cutting speeds but significantly influenced by the fiber orientation. Cutting forces presented smaller values in the fiber orientation of 0/180° and 15/165° but the highest one in 30/150°. The fracture mechanism of carbon fibers was studied in different cutting conditions such as 0° orientation angle, 90° orientation angle, orientation angles along fiber direction, and orientation angles inverse to the fiber direction. In addition, a prediction model on the cutting defects of carbon fiber reinforced plastic was established based on acoustic emission (AE) signals.

## 1. Introduction

As one of the most representative resin matrix composites, carbon fiber reinforced plastic (CFRP) composites have had an extensive application in the aviation field since the 1970s, such as the small-size cowling and air spoiler, the minor load-bearing structure of empennage, the main load-bearing parts of fuselage and airfoil [1,2,3], owing to their advantages of high specific strength and specific stiffness, good corrosion resistance and designability [4,5]. Therefore, CFRP has become the primary aeronautical structure material, replacing the aluminium alloy and high-strength steel gradually. There is a clear distinction between the cutting process of CFRP and metal materials on account of the anisotropy and heterogeneity of CFRP, making a new challenge in CFRP cutting [6]. The influence of anisotropy in CFRP cutting is mainly concentrated on obvious directionality, for instance, interlaminar bonding strength of CFRP laminates is only about 5%~20% of tensile strength along the fiber direction, making the interlaminar delamination more easily happen under the action of thrust forces. For another example, the material removal mechanism is completely different at various fiber orientations in the CFRP cutting process, leading to various acting forces in layers and poor machining quality. Consequently, processing defects are easily formed in CFRP machining process by reason of poor machinability and complex material removal rules caused by the characteristics of composite materials.

Wang DH *et al.* [7,8] made a systematic study on the material removal mechanism of unidirectional laminates in 1995. The main cutting force and radial cutting force were studied at four typical fiber orientations (0°, 45°, 90°, 135°) in orthogonal experiments and conclusions indicated that cutting forces of CFRP laminates presented significant differences under various fiber orientations while cutting forces of 0° and 135° fluctuated greatly and cutting forces of 45° and 90° presented stable conditions. Zhang LC *et al.* [9,10,11,12] established two-dimensional orthogonal cutting force models of CFRP with orientation angles along fiber direction (less than 90°) based on orthogonal cutting tests, without considering the orientation angles’ inverse fiber direction. Wang XM [9,10] studied cutting forces and surface topography of CFRP through orthogonal cutting tests which involved multifarious fiber orientation angles and conclusions were obtained that fiber orientation is the most significant factor affecting machinability due to obvious directionality of CFRP. Orthogonal cutting process using multiple cutting tools of different rake angles and relief angles was investigated by Arola D [13,14] so as to obtain the influence laws of tools parameters, cutting parameters, and fiber orientation on CFRP cutting forces and chip formation. An orthogonal cutting force model was established by Rao GVG [15,16,17] with the finite element method, explaining the formation of cutting force behavior and tearing defects. Cutting force increases linearly with the elevation of fiber orientation angle within the range of 0–90°.

In the matter of surface quality evaluation of CFRP, Sreejitha PS *et al.* [18] and Bhatnagar N *et al.* [19] made some analysis on chip formation of CFRP theoretically under conditions of orientation angles along fiber direction, inverse fiber direction, and parallel to fiber direction. Furthermore, cutting mechanisms and surface formation rules of CFRP were studied. Wang DH *et al.* [7] carried out orthogonal machining tests using CFRP unidirectional laminates and presented the cutting mechanism under different tool rake angles and fiber orientation angles.

Fiber prepreg molding technics are adopted to obtain the final CFRP structures in industrial production. The long fibers characteristically presented in commercial CFRP create distinct anisotropic characteristics. Besides, carbon fibers possess good hardness and high strength which are dozens of times of matrix materials, and the volume fraction of carbon fibers is larger than matrix materials. Therefore, carbon fibers consume more power and contribute more to the tool wear during the CFRP cutting process, which means that researches on cutting mechanisms and defect formation mechanisms of carbon fibers could provide important guidance to CFRP machining. As can be seen above, the main research in the literature is mainly focused on the machining situation of orientation angles along fiber direction and there are different statements on CFRP cutting forces variation laws in different orientation angles. Therefore, there is more challenge on cutting force behavior characteristics of CFRP. Material separation mechanisms and cutting mechanisms of carbon-fiber/epoxy prepreg sheets were investigated in this paper so as to make a study of the anisotropic behavior of CFRP. Fly cutting tests were adopted to study the mechanic variation laws. Furthermore, fracture mechanisms of carbon fibers both in macroscopic view and microscopic view were analyzed to obtain the defect mechanisms of unidirectional laminates under different fiber directions. Acoustic emission (AE) inspection was employed to realize the on-line monitoring and controlling of CFRP machining damages and the on-line monitoring model was established considering the significance of defect prevention.

## 2. Materials Preparation and Experimental Procedure

**Figure 1:**
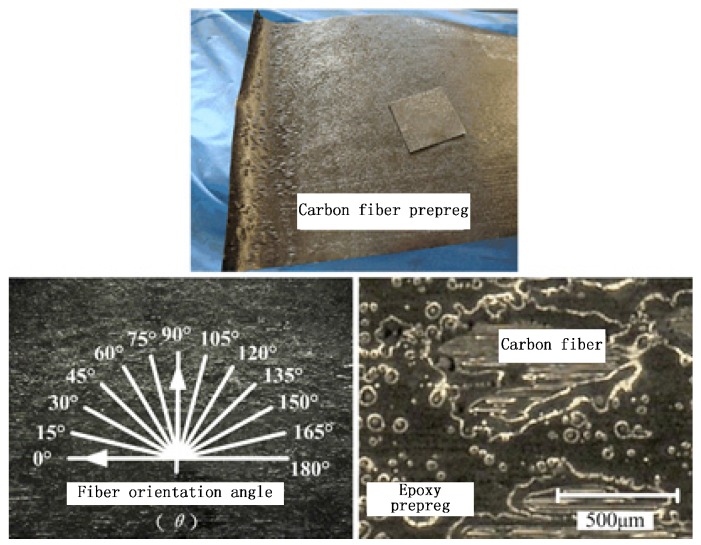
Carbon prepreg materials.

**Figure 2:**
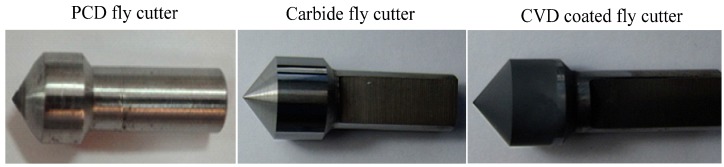
Cutting tools in experiments.

In this research, carbon fiber prepreg was used as the experimental material which was composed of T800 carbon fiber with fiber bundle of 12 K and ×850 epoxy, with the detailed physical and mechanical properties shown in Table 1. Carbon fiber prepreg was cut into pieces of 30 mm × 30 mm × 0.2 mm and single point cutting with different fiber directions was conducted as shown in Figure 1. As is shown in Figure 2, the cutting tools used in the experiments were three kinds of fly cutters with the same apex angle of 100° and different materials, namely, PCD material, CVD diamond thin film coated carbide material, and uncoated carbide material. The cutting edge radius of the PCD cutting tool is 0.5 mm while the CVD coated carbide tool and uncoated carbide tool show the values less than 60 μm.

**Table 1:** Physical and mechanical properties of T800 carbon fiber.

Material	Thermal Expansion Coefficient (K^−1^)	Thermal Conductivity (W·(m·K)^−1^)	Specific Heat (J·(kg·K)^−1^)	Density (g·cm^−3^)	Tensile Strength (MPa)	Tensile Modulus (GPa)
T800	−0.56 × 10^−6^	15.1	0.18	1.81	5490	294

**Figure 3:**
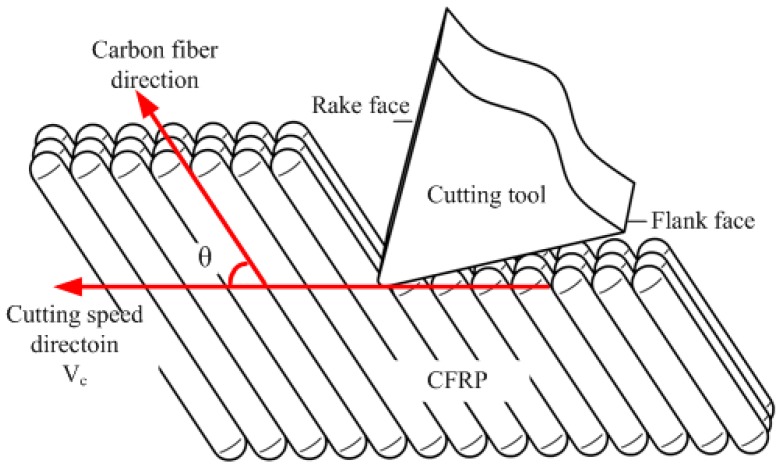
Scheme of fiber orientation angle.

Fiber orientation angle, defined as the angle between cutting speed (*V*_c_) and carbon fiber (pointing to the uncut material), is considered the critical factor influencing the machining mechanism of CFRP [7,9,19,20], shown in Figure 3.

**Figure 4:**
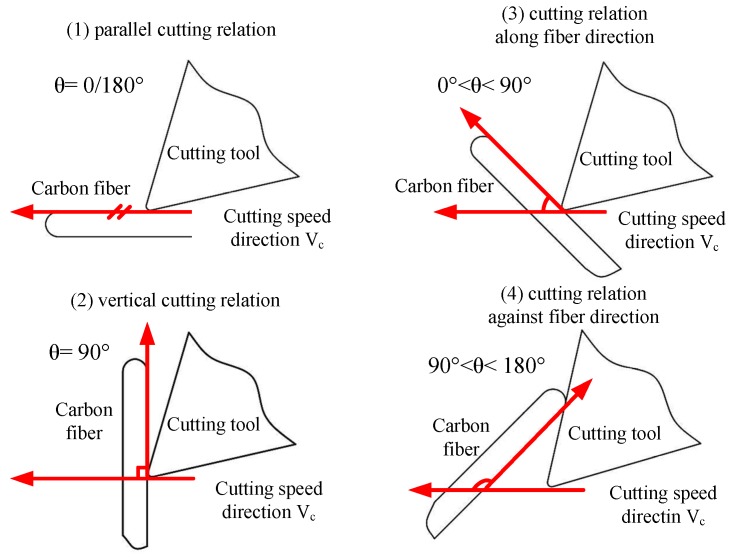
Cutting relationships between tool and carbon fiber.

Fiber orientation varies from 0° to 180°, which can be classified into four kinds of machining relations accordingly, shown in Figure 4.

(1) θ = 0°(180°), parallel cutting relation, with fiber and cutting speed in the parallel direction.
(2) θ = 90°, vertical cutting relation, with fiber and cutting speed in the vertical direction.
(3) 0° < θ < 90°, cutting relation along fiber direction, with fiber and cutting speed at an acute angle.
(4) 90° < θ < 180°, cutting relation inverse fiber direction, with fiber and cutting speed at an obtuse angle.

In the relationships of cutting tools and carbon fibers above mentioned, the cutting-off mode shows different conditions due to various geometrical positions, which include compressional fracture, bending fracture, and shear fracture. Considering the considerable differences existing in compressive strength, bending strength, and shear strength in diverse directions caused by CFRP anisotropy, the CFRP fracture property under different orientation angles is different. Therefore, there are significant differences in physical quantities of the cutting process such as cutting force, cutting temperature, surface quality, surface defect style, *etc.* The basic task in this research is to concentrate its efforts on the fracture property and fracture morphology of carbon fibers in the machining process, and thus the further study of the CFRP cutting mechanism.

**Figure 5:**
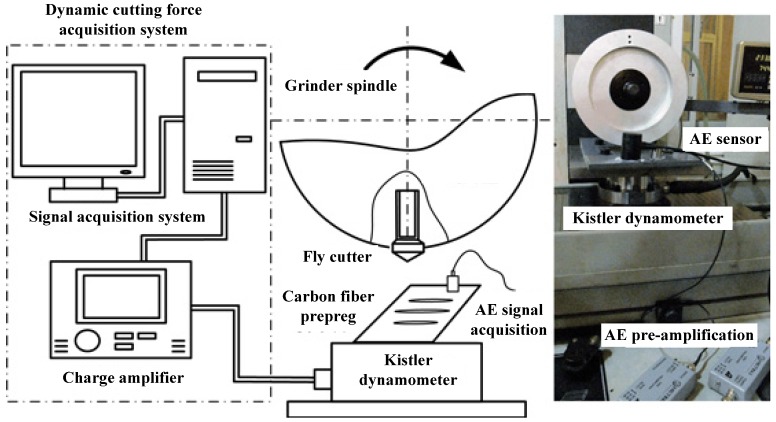
Configuration diagram of single point cutting of carbon fiber prepreg materials.

Figure 5 shows the configuration diagram of fly cutting of carbon fiber prepreg materials. Single point fly cutting method was adopted to study the carbon fiber fracture mechanism and fracture morphology under different fiber orientation angles. The fly cutting experiment was carried out on a KENT-KGS-1020 AH surface grinding machine. The workpiece material was fixed on the dynamometer. The charge signal achieved from dynamometer, which is based on principle of piezoelectric, was amplified by Kistler-5017-B amplifier, and cutting force signal was finally acquired through the computer acquisition system. Acquisition of acoustic emission was completed by an acoustic emission system with maximum 40 M Hz single channel digital sampling rate, which was composed of PAC- MICRO-II portable host and PAC-PCI-AE card. Moreover, a PAC-ISR3CA-HT sensor with a range of 10 KHz and 100 KHz frequency and a PAC-2/4/6-AST adjustable preamplifier with 20/40/60 db mode were used in this experiment.

**Figure 6:**
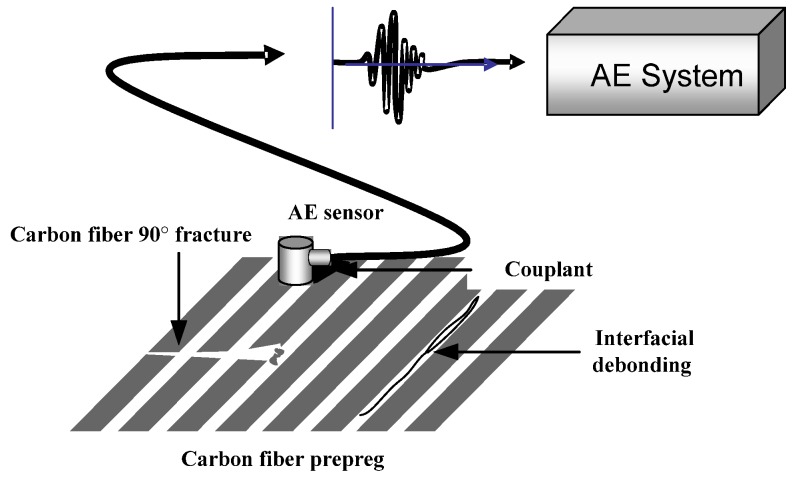
Acoustic emission monitoring of carbon fiber cutting.

Experimental parameters during carbon fiber prepreg fly cutting were shown in Table 2. As fly cutting is a process of cutting off continuous long fiber, the fiber orientation angles are shown as a pair of complementary angles during the fly cutting process, which means complementary fiber orientation angles are formed at the upper side and lower side of fracture surface. Therefore, the carbon fiber fly cutting test was divided into seven different orientation combinations of the angle between fiber direction and the cutting speed direction as below, 0/180°, 15/165°, 30/150°, 45/135°, 60/120°, 75/105°, 90/90°. Figure 6 shows the acoustic emission monitoring schematic diagram during carbon fiber prepreg cutting.

**Table 2:** Machining parameters in single point cutting prepreg materials.

Machining Parameters	Parameters Values
Fiber Orientation θ (°)	0, 90 (parallel, vertical) 15, 30, 45, 60, 75 (along fiber direction) 105, 120, 135, 150, 165 (inverse fiber direction)
Fiber style	T800
Cutting speed *V*_c_ (m/min)	50, 100, 150, 200, 250, 300
Fly cutting depth (μm)	20

## 3. Results and Discussion

### 3.1. Effect of Fiber Orientation on Cutting Forces

**Figure 7:**
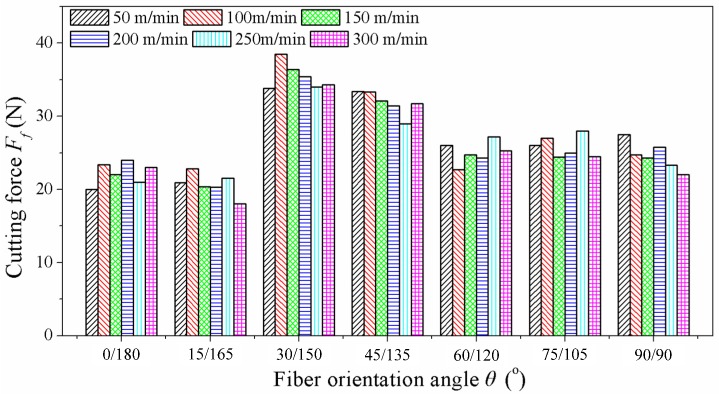
Cutting force results of PCD tool.

Figure 7 demonstrates the cutting force *F*_f_ (along the cutting speed) of carbon fibers cut in different fiber orientation angles θ using the PCD fly cutter. It can be seen that lower cutting forces of carbon fibers are obtained while the impact of fiber orientation angles is significant and the effect of cutting speeds can be ignored. In other words, the cutting fracture of carbon fibers occurs easily. Moreover, the cutting forces of carbon fibers are not affected by the cutting speed *V*_c_, keeping a similar hard-brittleness fracture mechanism, while the effect of orientation angles on the cutting forces cannot be neglected. The cutting forces produced in 0/180° and 15/165° are significantly lower than the other cutting forces. The cutting forces *F*_f_ have a rapid growth in the 30/150° orientation angle and then reduce with the elevation of the orientation angle.

**Figure 8:**
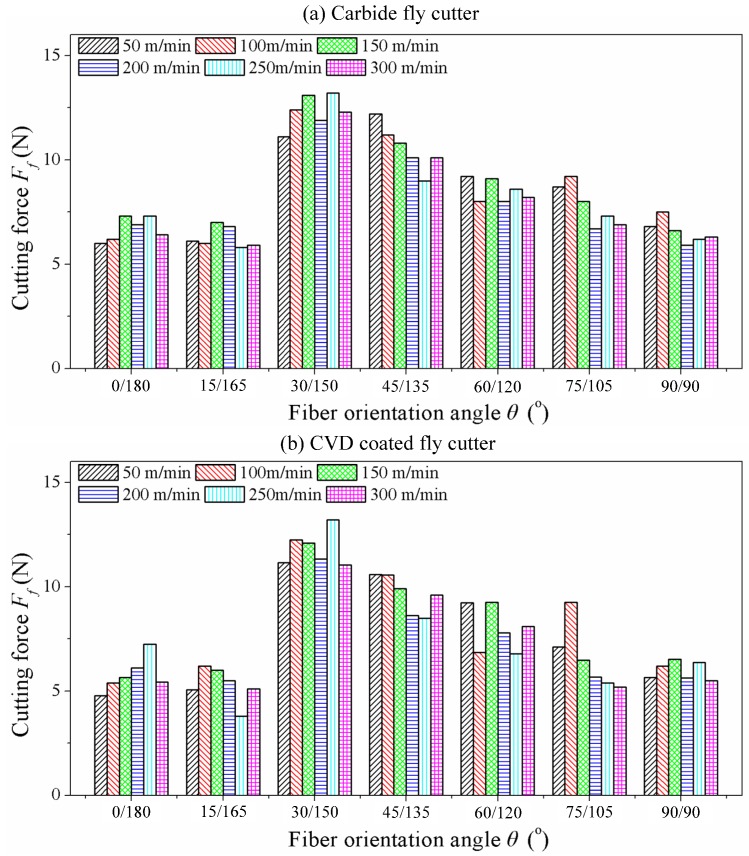
Cutting force results of carbide tool and diamond coating tool. (**a**) Carbide fly cutter; (**b**) CVD coated fly cutter.

As can be seen from Figure 8 showing the cutting forces *F*_f_ in different fiber orientation angles with carbide fly cutter and CVD coated carbide fly cutter, conclusions can be drawn that the laws of cutting are consistent with the PCD cutting tool. The cutting force *F*_f_ presents a lower value when the fiber orientation angle θ is less than 30° while *F*_f_ decreases with the increase of θ when the orientation angle θ is equal or greater than 30°. Furthermore, the cutting forces produced by the carbide fly cutter and CVD coated fly cutter are lower than the PCD fly cutter apparently owing to the blunt cutter tip of PCD tool and thus removing a greater volume of material at the same cutting depth. By comparison, uncoated carbide fly cutter can produce greater cutting forces than CVD coated carbide fly cutter, which can be attributed to the smaller friction coefficient of CVD diamond coating.

### 3.2. Effect of Fiber Orientation on Cutting Fracture

**Figure 9:**
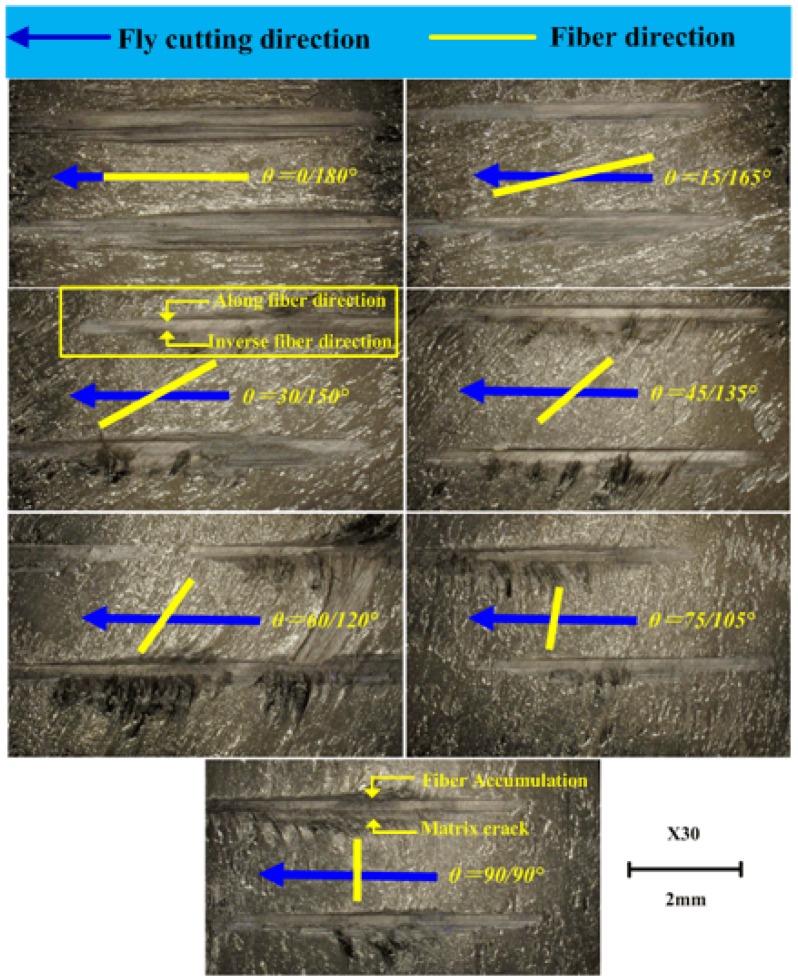
Macroscopic fracture morphology in prepreg fly cutting with PCD tools.

As a layer of CFRP unidirectional laminates, carbon fiber prepreg can easily produce manufacturing deficiency without enough resin support protection in fly cutting process. It is very similar to the cutting process of CFRP unidirectional laminates. Therefore, the defect formation mechanism during CFRP unidirectional laminates cutting can be figured out through the analysis of fracture defects of carbon fiber prepreg in fly cutting tests. Figure 9 shows macroscopic fracture morphology of T800 carbon fiber prepreg in fly cutting process by PCD tool in different fiber orientation angle θ. It can be concluded that the carbon fibers have the characteristic of significant anisotropy and different fracture features are displayed as fiber direction angle θ varies within the range of 0~180°. It has also shown that the orientation angles of 0/180° and 15/165° can produce the best fracture quality with a relatively smooth fracture at both upper side and lower side of fracture surface. As the main failure mode in 0/180° and 15/165° is resin bond failure rather than carbon fiber fracture, the macroscopic fracture morphology is smooth with undamaged matrix material. In the orientation angles of 30/150°, 45/135°, and 60/120°, the upper side of fracture surface shows different morphology from the lower side of the fracture surface. Accumulation of fibers which are not cut off appear at the upper side of fracture surface with more evident accumulation happening at smaller fiber orientation angle. Matrix cracking appears at the lower side of fracture surface with more evident tearing phenomenon at greater fiber orientation angle. That is to say, carbon fiber is hard to be cut off and fiber accumulation appears easily at the fracture surface at the range of 15° < θ ≤ 60°, while matrix material is easily to produce cracks at the range of 120° ≤ θ < 165°. In the orientation angles of 75/105° and 90/90°, fiber accumulation at the upper side of fracture surface becomes inconspicuous with the θ ranging from 75° to 90°, while the matrix cracks at the lower side of fracture surface decrease with the θ ranging from 90° to 105°. In conclusion, the cutting along fiber direction and inverse fiber direction appears respectively at the upper side and lower side of fracture surface, as in shown in Figure 9. In addition, relatively smooth fractures are obtained along the fiber direction with fiber accumulation appearing at a certain fiber orientation angles, while rough fracture is acquired along the inverse fiber direction with matrix cracks occurring commonly. Considering the need of processing quality evaluation during carbon fiber machining, the splintering extent of matrix material can be used as the quantified indicator.

**Figure 10:**
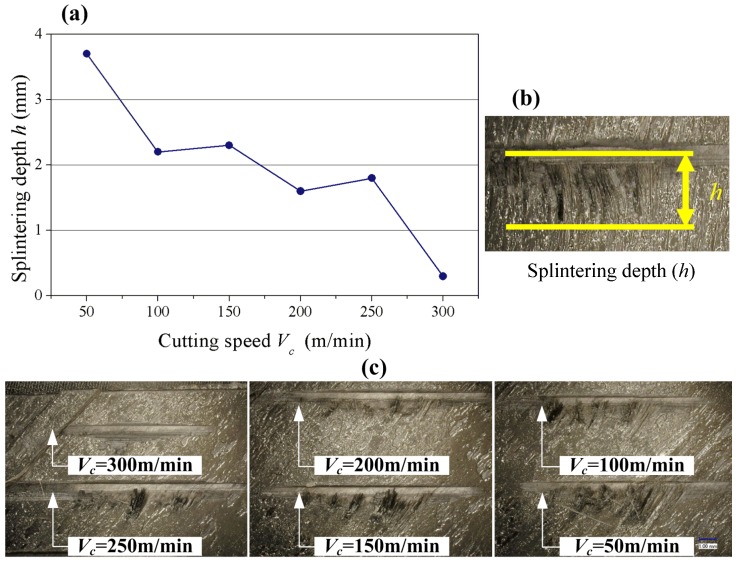
Effects of cutting speed on splintering depth in single point cutting of carbon fiber (45/135°) with PCD tools. (**a**) Line chart; (**b**) Splintering depth; (**c**) The fracture morphology.

As is shown in Figure 10b, *h* is defined as the maximum splintering depth in the fly cutting of carbon fiber prepreg during which *h* can be applied to analyze the effect of different cutting speeds *V*_c_ on machining quality of carbon fiber. Figure 10c shows the fracture morphology of T800 carbon fiber in the orientation angle of 45/135°: As can be seen from Figure 10 that splintering depth varies significantly with the variation of cutting speed at the lower side of fracture surface. Figure 10a shows the curve of splintering depth changing with cutting speed. Conclusions are drawn that high speed cutting can significantly decrease the splintering depth of matrix material so as to achieve better fracture morphology. It is due to the fact that there is not much time for tearing defects to spread at higher cutting speed, which results in decreasing splintering depth.

**Figure 11:**
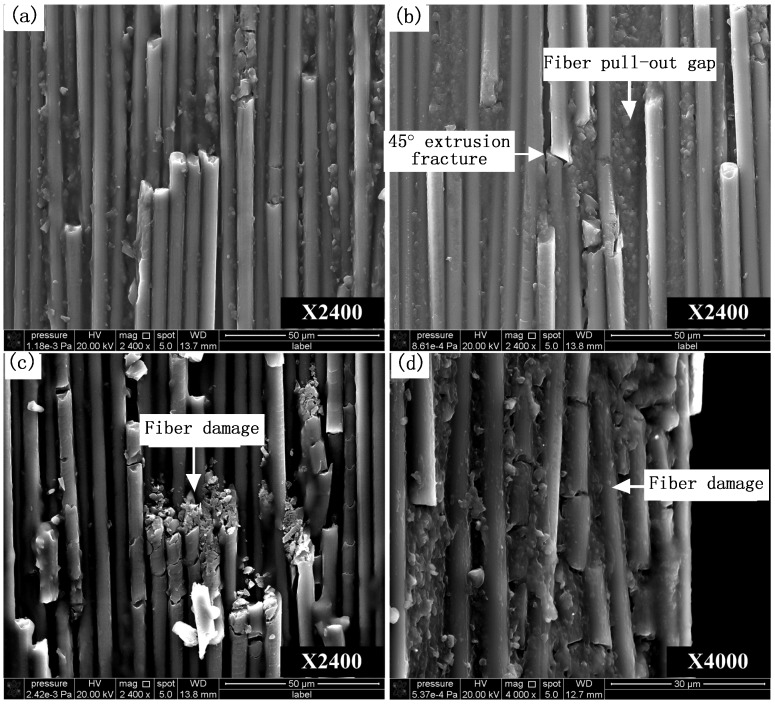
Fracture topography SEM photos of carbon fiber with 0° orientation angle. (**a**–**c**) 0° orientation angle with 2400X magnification; (**d**) 0° orientation angle with 4000X magnification.

Figure 11 shows the microscopic morphology of surface defects in carbon fiber cutting in the fiber orientation angle of 0(180)° which is featured by 45° oblique fracture caused by extrusion. As can be seen from Figure 11 that smooth microscopic morphology is obtained and the main surface defects are fiber pull-out gap and fiber damage. Fiber pull-out gap is the surface gap due to the fibers debonding from matrix material, and fiber damage is owing to continuous long fibers being broken into intermittent short fibers. The formation of the main surface defects is because the fiber cut-off in 0° fiber orientation is mainly along the fiber direction which makes a part of fibers losing the enhancement effect on matrix material.

**Figure 12:**
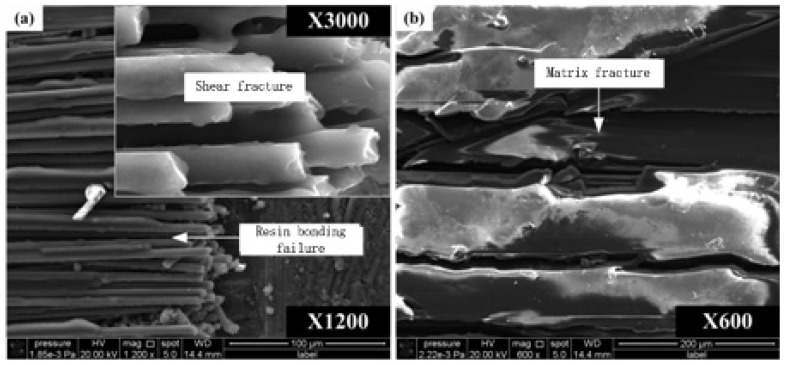
Fracture topography SEM photos of carbon fiber with 90° orientation angle. (**a**) 90° orientation angle with 1200X magnification; (**b**) 90° orientation angle with 600X magnification.

SEM (Scanning Electron Microscope) photos of fracture topography in the fiber orientation angle of 90° during carbon fiber fly cutting are shown in Figure 12. Level fracture caused by shear is the main feature of carbon fiber fractures, which suggests that the cut-off type of carbon fiber is shear fracture along 90° orientation angle. The main forms of surface defects are matrix crack and resin bond failure which lead to fiber deprived of support from the matrix material. Because the shear strength of carbon fiber in the 90° orientation angle is much bigger than interlaminar tensile strength, interface between carbon fiber and the resin matrix will be destroyed firstly and generate the cracks, which makes carbon fiber near cracks lose support from the matrix material. Cracks can be easily spread to the matrix material through these long carbon fibers which are not supported by matrix material, eventually resulting in matrix material splintering. Even if there is no extensive matrix material splintering, resin bond failure has happened near the fracture surface, losing the protection of carbon fibers.

**Figure 13:**
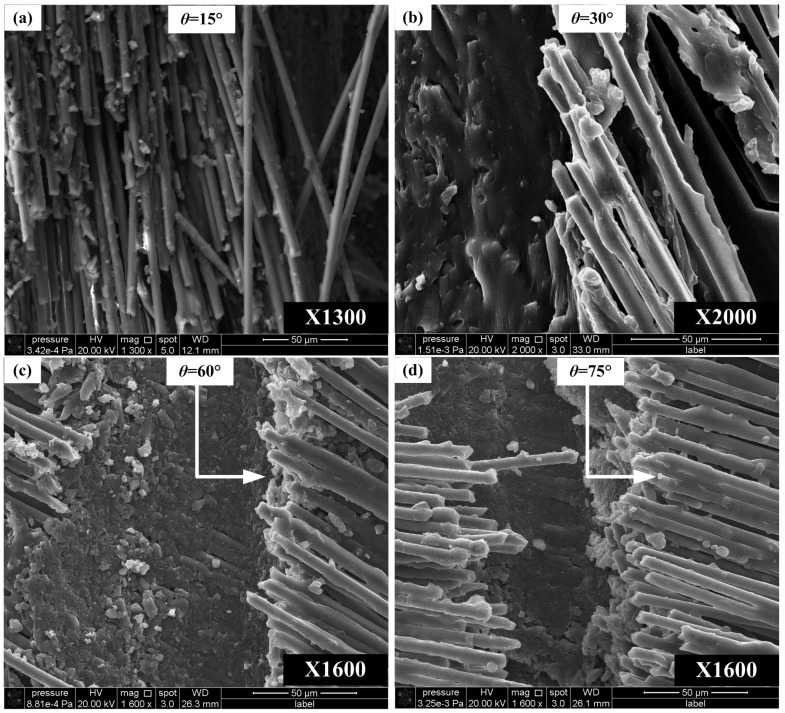
Fracture topography SEM photos of carbon fiber with orientation angles along fiber direction. (**a**) 15° orientation angle with 1300X magnification; (**b**) 30° orientation angle with 2000X magnification; (**c**) 60° orientation angle with 1600X magnification; (**d**) 75° orientation angle with 1600X magnification.

Figure 13 shows the carbon fiber fracture topography in the fiber orientation angle ranging from 0° to 90°. It can be concluded that the fracture surface mainly consists of resin matrix with some fiber fractures. Resin matrix provides good protection of carbon fibers and the relatively smooth microscopic morphology can be observed. Moreover, the fracture surface has a tendency of getting rougher with the increase of θ. Fiber accumulation, *i.e.*, burr defects, easily occurs at the range of 0° < θ < 30°, while matrix crack and resin bond failure occur easily at the range of 60° < θ < 90°.

**Figure 14:**
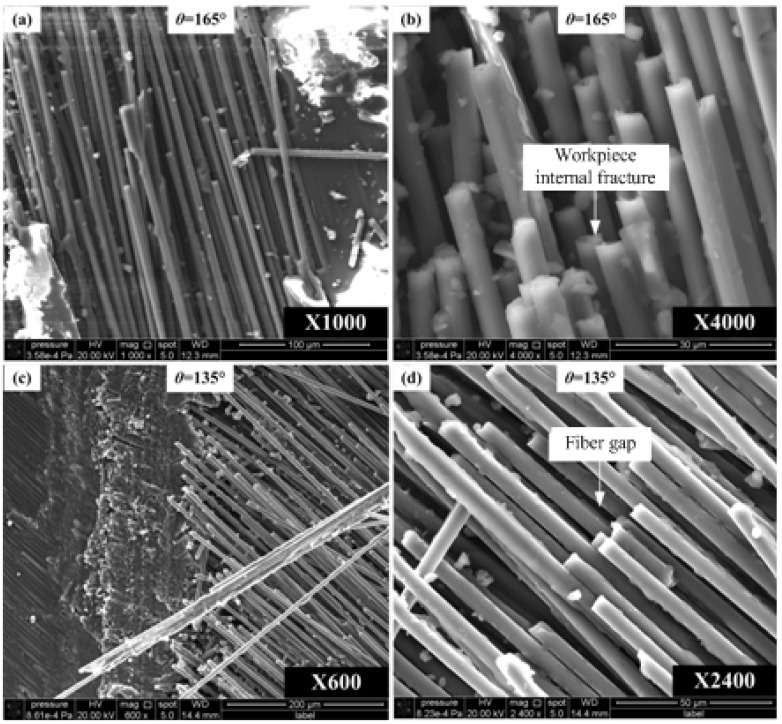
Fracture topography SEM photos of carbon fiber with orientation angles inverse fiber direction. (**a**) 165° orientation angle with 1000X magnification; (**b**) 165° orientation angle with 4000X magnification; (**c**) 135° orientation angle with 600X magnification; (**d**) 135° orientation angle with 2400X magnification.

The fracture topography in the fiber orientation angle ranging from 90° to 180° is shown in Figure 14. The fracture surface is made up of uneven fiber fractures which are smooth but have uneven length with poor surface roughness. Resin cracking is severe and common with carbon fibers totally losing support. Level fracture caused by bending and breaking is the main feature of fracture surface. Therefore, the main surface defects are featured by matrix crack and resin bond failure which result in carbon fibers losing the protection from matrix materials. Furthermore, workpiece inner fractures can be generated at the bending and breaking point of carbon fibers, thus resulting in fiber gaps at the surface layer. Because cutting direction is the inverse of fiber direction, carbon fiber cracks cannot be directly sheared or extruded under the effect of the fly cutter. Cracks are generated by the interface debonding, leading to losing its support from matrix. Then, cracks are spread into the workpiece along the long fibers, which eventually leads to matrix splintering and carbon fiber tearing inside workpiece.

### 3.3. Cutting Defects Prediction Modeling of CFRP Based on AE Signals

Because of the significant anisotropy, it is easy to have cutting defects during the machining process of CFRP. CFRP machining is generally the finishing process after molding and before assembly, thus machining defects can make CFRP components become the rejects before final assembly. Therefore, it can strengthen the on-line monitoring and controlling of CFRP machining damages by introducing the AE inspection [21,22,23,24]. It is important to establish the quantitative relationship model between cluster parameters of splintering defects in AE monitoring and actual splintering depth *h* of prepreg material, *i.e*., the prediction model of CFRP machining defects based on AE signals, which can provide guidance for predicting defects during CFRP machining.

**Figure 15:**
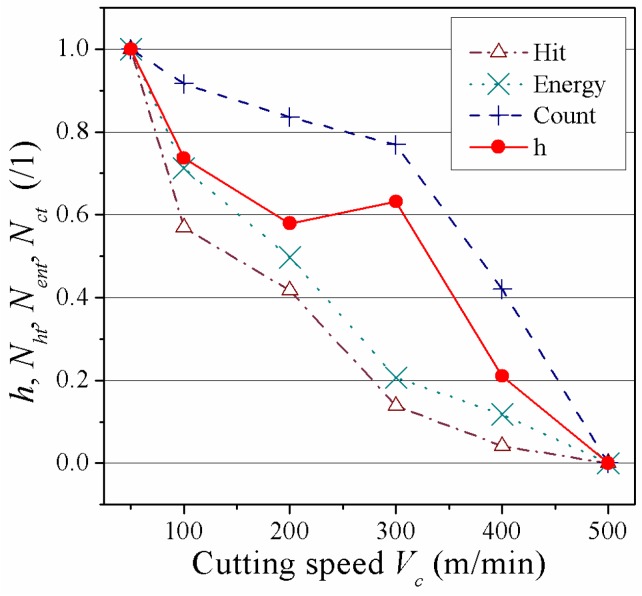
Relation of AE cluster parameters and splintering depth.

It is AE statistics of splintering clusters at different cutting speeds during T800/X859 prepreg fly cutting by CVD coated carbide cutting tool shown in Table 3. It can be seen that splintering depth *h* changes with the variation of cutting speed ranging from 50 to 500 m/min, and the hit, energy, and ringing count of AE splintering cluster with about 76 kHz peak frequency also would change. Figure 15 is derived based on these statistics. A linear model as shown in Equation (1) is established to predict splintering depth *h*, among which *N_ent_* represents energy count; *N_ct_* is ringing count; *N_ht_* is on behalf of hit count; *L* is cutting length, *a*, *b*, *c* are related linear coefficients.

(1) $$h=a\cdot \frac{N_{ht}}{L}+b\cdot \frac{N_{ent}}{L}+c\cdot \frac{N_{ct}}{L}$$

By substituting the statistics shown in Table 3 into Equation (1), prediction model based on AE signal could be established for splintering defect of carbon fiber prepreg, as shown in Equation (2). Moreover, as prepreg fly cutting is similar to machining of CFRP unidirectional laminates, Equation (2) could be used for splintering defect prediction of CFRP unidirectional laminates at the entrance and exit.

(2) $$h=136.3584\frac{N_{ht}}{L}-4.064\frac{N_{ent}}{L}+4.2544\frac{N_{ct}}{L}$$

**Table 3:** AE statistics of splintering cluster under different cutting speeds.

Cutting Speed (m/min)	Hit (count)	Energy (count)	Ringing (count)	Average Peak Frequency (kHz)	Average Power (count)	Splintering Depth (mm)
50	72	2023	1624	75.81	68	1.9
100	31	841	605	75.81	68	1.4
200	14	506	360	75.14	68	1.1
300	4	318	195	76.00	75	1.2
400	3	39	28	76	77	0.4
500	0	0	0	0	0	0

Table 4 shows the comparison between the prediction based on Equation (2) and true splintering depth. It can be seen that the deviation is less than 15%. 

**Table 4:** Prediction results of splintering depth based on AE.

Comparison Program	Comparison Results						
Prediction Value (μm)	1913	1322	1266	1081	495	0
Test value (μm)	1920	1410	1100	1200	430	0
Absolute error (μm)	7	88	166	119	65	0
Relative error (%)	0.3	6.2	15.1	9.9	15.1	0

## 4. Conclusions

In this study, the effects of fibre orientation on cutting forces and fracture topography of carbon fiber were investigated using three kinds of cutting tools. Moreover, prediction modeling of cutting defects was proceeded so as to monitor the machining process online. The conclusions can be drawn as follows:

1. The effect of cutting speeds on cutting forces of carbon fibers can be ignored while the influence of orientation angles is obvious.
2. The cutting forces produced in 0/180° and 15/165° are significantly lower than the cutting forces in other orientation angles using three kinds of fly cutters in prepreg cutting. The cutting forces F_f_ have a rapid growth in the 30/150° orientation angle and then reduce with the elevation of the orientation angle.
3. Fiber accumulation is formed easily at the fracture surface at the range of 15° < θ ≤ 60°, while matrix material is easily to produce cracks at the range of 120° ≤ θ < 165°. High speed cutting decreases the splintering depth greatly so as to achieve better fracture morphology.
4. In the carbon fiber cutting with the fiber orientation of 0(180)°, the smooth microscopic morphology can be obtained and the main surface defects are fiber pull-out gap and fiber damage.
5. In the carbon fiber cutting with the fiber orientation of 90°, level fracture caused by shear is the main feature of carbon fiber fractures, which indicates that the cut-off mode of carbon fiber is shear fracture along 90° orientation angle. The primary forms of surface defects are matrix crack and resin bond failure which could make carbon fiber lose support from the matrix material.
6. In the carbon fiber cutting with the orientation angles along fiber direction (0° < θ < 90°), the fracture surface is mainly composed of matrix materials with some fiber fractures. The relatively smooth microscopic morphology can be obtained and the fracture surface becomes more rough with the elevation of θ. Fiber accumulation occurs easily at the range of 0° < θ < 30° while matrix cracks and resin bond failure appear at the range of 60° < θ < 90°.
7. In the carbon fiber cutting with the orientation angles inverse fiber direction (90° < θ < 180°), the fracture surface is made up of fiber fractures which are smooth but have an uneven length with poor surface roughness. Level fracture caused by bending and breaking is the main feature of fracture surface. Therefore, the main surface defects are featured by matrix crack and resin bond failure which result in carbon fibers losing support from the matrix material. In addition, workpiece inner fracture is generated at the bending and breaking point of carbon fibers, thus causing fiber gap at surface layer.
8. A prediction model on the cutting defects of CFRP is established based on acoustic emission signals, which can be used to monitor the processing defects online.
